# Trajectories of end-of-life medical and long-term care expenditures for older adults in Japan: retrospective longitudinal study using a large-scale linked database of medical and long-term care claims

**DOI:** 10.1186/s12877-021-02215-9

**Published:** 2021-06-30

**Authors:** Emi Teraoka, Susumu Kunisawa, Yuichi Imanaka

**Affiliations:** Department of Healthcare Economics and Quality Management, Graduate School of Medicine, Kyoto University, Yoshida Konoe-cho, Sakyo-ku, Kyoto City, 606-8501 Japan

**Keywords:** End-of-life expenditure, Long-term care, Geriatric care

## Abstract

**Background:**

An accurate understanding of the current state of end-of-life care is important for healthcare planning. The objectives of this study were to examine the trajectories of end-of-life medical and long-term care expenditures and associated factors.

**Methods:**

This was a retrospective longitudinal study using a large-scale linked database of medical and long-term care claims—National Health Insurance, Advanced Elderly Medical Insurance, and long-term care insurance—covering Prefecture A in Japan. Patients aged ≥70 years who died between April 1, 2016, and March 31, 2017, were included (*N* = 16,084 patients; mean age = 85.1 ± 7.5 years; 7804 men (48.5%) and 8280 women (51.5%)). The outcome measures were medical expenditures (inpatient, outpatient, and prescription), long-term care expenditures, and total healthcare expenditures (the sum of medical and long-term care expenditures) during the 60 months before the date of death. We calculated each patient’s monthly medical and long-term care expenditures for 60 months before the date of death and applied group-based trajectory modeling to identify distinct trajectories. Factors associated with spending trajectories were examined via multinomial logistic regression analyses. Explanatory variables included age, sex, diseases, and the medical services used.

**Results:**

We identified six distinct spending trajectories for the total healthcare expenditures: high persistent (45.6%), medium-to-high persistent (26.1%), early rise then high persistent (9.8%), late rise (6.4%), low persistent then very late rise (i.e., when spending starts increasing later than “late rise”; 6.4%), and progressive increase (5.7%). Factors associated with the high-persistent trajectory were chronic illnesses, various organ failures, neurodegenerative diseases, fractures, and tube feeding. The trajectory pattern of medical expenditures was similar to that of total healthcare expenditures; however, a different pattern was seen for long-term care expenditures.

**Conclusions:**

Regarding combined medical and long-term care spending of the last 5 years, most patients belonged to a pattern in which the healthcare expenditures remained high, and a combination of multiple factors contributed to these patterns. This finding can offer healthcare providers a longer-term perspective on end-of-life care.

**Supplementary Information:**

The online version contains supplementary material available at 10.1186/s12877-021-02215-9.

## Background

End-of-life care is an important part of healthcare. As older people are more likely to die than their younger counterparts, an increase in the elderly population means an increase in the number of deaths. Because Japan has the highest rate of aging in the world and is the fastest aging country, the size of its end-of-life population is increasing. Notably, this increase is now being recognized as a social issue, termed the arrival of the “frequent death society.” [1] Specifically, the number of deaths per year in Japan is estimated to peak around 2040, with an estimated increase of 360,000 deaths between 2015 and 2040. Meanwhile, Japan’s average life expectancy is also increasing alongside an increase in elderly deaths. In 2018, 75.8% of deaths occurred in individuals ≥75 years of age and 47.6% in individuals ≥85 years of age [2]. These demographic changes are placing pressure on Japan’s end-of-life care, and responses to these pressures are needed in various sectors, especially healthcare [1]. To ensure future system sustainability, the allocation of resources to end-of-life care and the management of quality are thus recognized as urgent social issues for insurers and healthcare providers [3].

Empirical knowledge and previous studies have suggested that the trajectory of end-of-life disability depends on the disease that causes impairment [4, 5] Understanding this trajectory can provide information early end-of-life care discussions between patients and healthcare providers, which can promote positive end-of-life care experiences for patients [6]. Helpful to note here is that some studies have demonstrated that patients’ healthcare expenditures increase in the period prior to their death —an indicator of the increased demand for healthcare [7–9], .Two notable studies [10, 11] adapted Nagin et al.’s (2010) group-based trajectory modeling [12] to end-of-life trajectories. Meanwhile, Gill and colleagues used group-based trajectory modeling to determine the pattern of decline in physical function during the year before death [10]. Their results showed that nearly half the patients had persistent or slowly progressive disability. Davis and colleagues used Medicare data to conduct a similar analysis of end-of-life healthcare expenditures [11]. They identified four trajectories of healthcare expenditures during the final year before death. The most frequent trajectory was for the expenditures to be high throughout the period (high persistent); this was associated with the presence of multiple chronic diseases and had the greatest impact on end-of-life healthcare expenditures among all other trajectories. Although the numbers of trajectories derived in these two studies differed, there was a common outcome: the most frequent pattern was sustained high. Thus, physical disability was inferred to be associated with Medicare expenditures and most patients had high healthcare demands for more than 1 year during the end-of-life period. The results of these studies provided novel insights into the trajectories of end-of-life expenditures. However, what was not clear was the onset of the high degree of disability or healthcare expenditures. Additionally, the contrasting factors in these two studies require clarification, and Medicare expenditures may not reflect all of patients’ healthcare demands.

Moreover, previous studies only addressed when an increase in end-of-life healthcare demand began. Seshamani and Gray, who examined longitudinal data over a 29-year period in England, reported that inpatient expenditures began to increase slightly from 15 years before death [9]. In addition, they pointed out that the yearly costs nearly doubled over 7 years, from 15 to 8 years before death, then nearly doubled again 3 years later in the fifth year before death, and 2 years later in the third year before death. Eventually, the costs increased more than seven-fold from the third year to the year before death. Lunney and colleagues compared activities of daily living between deaths and survivors during the last 3 years and noted that functional declines began 3 years before death [13]. French and colleagues compared healthcare expenditures one and 3 years before death in nine countries around the world and suggested that chronic illness affects end-of-life expenditures over more than 1 year [14]. The role of long-term care insurance in end-of-life healthcare expenditures was also suggested in that study. They noted that, because countries with stronger long-term care sectors tend to have less acute care spending, this might indicate some substitution of services across the two sectors. Therefore, it is suggested that a period of more than 1 year be considered to better understand end-of-life trajectories. Furthermore, expenditures may begin increasing 3 years before death. It is also necessary to consider long-term care insurance and medical care expenditures.

In Japan, all citizens are covered by public health insurance and are required to register with insurance based on their employment status [15]. National Health Insurance covers self-employed people and retirees, and the Late-stage Elderly Health Insurance covers people aged ≥75 years. Both are managed by local governments. Most people aged ≥65 years are covered by one of these schemes. Japan’s long-term care insurance, introduced in 2000 [3, 16], ,is a social insurance scheme independent of medical insurance such as National Health Insurance, the Late-stage Elderly Health Insurance and other employees’ insurances. Local governments are insurers, and citizens aged ≥65 years (or ≥ 40 years with a specific disease) are insured. Services include home visiting care, day care, institutional care, and other services that are provided across seven levels of care. ﻿ The seven care-level categories are further divided into two types: care levels 1–5 for individuals with greater levels of disability needing help with the basic activities of daily living (ADL). The “support required” levels 1 and 2 for individuals who can live independently, but are afraid of needing care and require assistance for the instrumental activities of daily living (IADL). In 2014, the long-term care expenditures were about one-fourth of the national medical expenditures (all ages) [17] but their growth rate exceeded the medical expenditures and is expected to increase further [18]. One of the objectives of introducing long-term care insurance in Japan was to reduce healthcare expenditures by providing home care and decreasing the number of “social hospitalizations” that used medical resources for long-term care purposes [19, 20]. In a system like Japan’s, where public health insurance and long-term care insurance are compatible, coordination between medical services and long-term care services must be paid attention to when considering a healthcare delivery system for the elderly.

Our study objective was to clarify the trajectories of medical and long-term care expenditures for older adults (aged ≥65 years) during the final 5 years prior to death, and to investigate the interrelationship of medical care and long-term care and the factors associated with these trajectories. As prior literature suggests that a large increase occurs 3 years before death, we hypothesized that by examining medical and long-term care expenditures for 5 years, we could consider the starting point for an increase in end-of-life expenditures, and the interaction between medical and long-term care expenditures. For healthcare providers, a better understanding of the trajectories and needs of end-of-life medical and long-term care may be valuable for the development of care. We examined end-of-life expenditures related to both medical and long-term care, and we did so over a longer period than many previous studies on end-of-life care expenditures. Thus, this study provides specific information concerning the improvement of end-of-life care provision and elucidates what needs to be focused on, the time period covered, and the current state of medical and long-term care services allocations.

## Methods

### Data source and patients

This retrospective longitudinal study used linked claims databases for National Health Insurance, Advanced Elderly Medical Insurance, and Long-term care Insurance that covered Prefecture A in western Japan. The medical claims data (from the National Health and Advanced Elderly Medical Insurance databases) included inpatient, outpatient, and prescription claims, but not dental claims. Data with billing dates from April 2010 to March 2015 were extracted and examined.

The data eligible for inclusion in the analysis were for patients who died between April 1, 2014, and March 31, 2015, and who were aged ≥70 years at their death, excluding 15 individuals with data inconsistencies (*N* = 16,084).

### Main outcome measures

The outcome measures were medical expenditures (inpatient, outpatient, and prescription), long-term care expenditures, and total healthcare expenditures (the sum of medical and long-term care expenditures) during the 60 months before the date of death. Some hospital costs are from both high-output hospitals and those that employ the DPC/per-diem payment system (PDPS) [21]. Based on the Organization for Economic Co-operation and Development’s (OECD) System of Health Accounts (SHA) 2011 [22], medical expenditures were classified as curative care, rehabilitative care, ancillary services, and medical goods. Dental care was not included. Using SHA 2011 coding, The long-term care expenditures in this study included everything classified by SHA 2011 coding as HC.3Long-term care (health), part of HC.1.4Home-based curative care(i.e. part of home visiting nurse) in HC.1Curative care, and part of HCR.1 Long-term care (social) (i.e. home visiting care, day care, and institutional care for patients with lower two care levels) classified in HCR Health care-related classes. The categories for preventive care and governance and health systems were not included in the analysis [23]. Services included in Japanese health insurance and long-term care insurance and their correspondence with those included in SHA 2011 are shown in the Additional File Table S1. Monthly and total expenditures for each patient for the 60 months prior to death were calculated, with the date of death used as the starting point.

### Explanatory variables

The explanatory variables used were age, sex, six medical services, and 21 diseases. Age—defined as the age at death—was treated as a continuous variable, whereas the other variables (service use and diseases) were all binary. For medical services, data related to six types of service were extracted from the medical claims: home medical care by a physician, dialysis, tube feeding, ventilation, hospice, and death at home/long-term care facility. These explanatory variables were selected from the data to be in line with previous studies that demonstrated that they affected end-of-life healthcare expenditures [11, 24] The 21 diseases were extracted from all the diseases listed in the claims data for inpatient and outpatient care and are linked with their International Classification of Diseases codes in Additional file Table S2.

The selected diseases were chronic diseases, chosen from among the most common outpatient and inpatient diseases reported in the Japan Ministry of Health Labor and Welfare’s 2014 Patient Survey [25], the top reasons for the introduction of long-term care insurance reported in the Ministry’s National Lifestyle Survey 2013 [26], and the leading causes of death in the Ministry’s published 2015 statistics [27]. Hematologic malignancies were separated from the other malignancies because they tend to be more expensive [28]. Information on diseases was defined as an explanatory variable by identifying disease names that appeared more than once in the medical claims during the last 5 years of life because it was inherently difficult to identify exact dates of onset of chronic disease. Healthcare service utilization was identified from medical claims during 1 year before death.

### Statistical analysis

The descriptive statistics included total values and percentages. We separately performed statistical analyses for the medical, long-term care, and medical and long-term care expenditures. Means, standard deviations, and maximum and minimum expenditures were calculated monthly and for the entire 12 months and 60 months prior to death. We also included descriptive statistics for participants with no costs over the last 5 years. The relationship between the individual totals of medical and long-term care expenditures for the last 60 months and their cumulative ratio are described as a Pareto chart.

We applied group-based trajectory modeling [12] to identify distinct trajectories for the medical and long-term care expenditures over the 60 months before the date of death. The expenditure data distributions were natural logarithmically transformed to correct for distortions (assigning zero as the transformed value if a monthly expenditure value was zero). We constructed models after determining the number of groups (from 2 to 8) and the combination of trajectory shapes (intercept-only, linear, quadratic, cubic, quartic, and quintic) and investigated them. In the model selection, we identified the optimal combination of the numbers of groups and orders using the Bayesian information criterion, and we excluded the models in which the smallest estimated proportion of a sample assigned to a certain group was < 5%. The accuracy of the models was evaluated using four diagnostic measures: the average posterior probability of assignment for each group (0.7 or higher), the odds of correct classification (5.0 or higher), the proportion of a sample assigned to a certain group is close to the proportion estimated from the model, and 98% confidence intervals for the estimated proportion (Additional file Text S3). Group-based trajectory modeling was performed for all the samples and subgroups of males and females.

After determination of spending trajectories, descriptive statistics of participants’ characteristics were summarized using total values and percentages; and expenditures were summarized using means, standard deviations, maximum–minimum, and percentages of total expenditures. The number of participants assigned to each trajectory groups was cross-tabulated for the combination of three expenditure categories (i.e., total of medical and long-term care vs. medical, total of medical and long-term care vs. long-term care, medical and long-term care).

Multiple multinomial logistic regression analyses were used to examine the factors associated with the spending trajectories that resulted from the group-based trajectory modeling. Two models were constructed with two different combinations of explanatory variables. In Model 1, age, gender, 6 medical services, and 21 diseases were adopted as explanatory variables. In Model 2, age, sex, 6 medical services, and the number of diseases were adopted as explanatory variables.

The monthly trends of per patient expenditures were captured by medical and long-term care spending trajectories.

Stata version 15.1 (Stata Corp. College Station, TX) was used for statistical analyses. Two-tailed *p*-values < .05 were considered significant. Group-based trajectory modeling was performed using the STATA “traj” plugin. Final expenditure estimates were converted to US dollars using the average monthly purchasing power parity rate from April 2010 to March 2015 based on OECD data (US $1.00 = 92 Japanese yen).

## Results

### Patients’ characteristics

Patients’ characteristics are summarized in Table 1. The sex distribution was nearly equal, with 51.5% females and 48.5% males. The mean age (at death) was 85.1 ± 7.5 years. Most had been hospitalized. The most common diseases were chronic respiratory failure (78.0%), hypertension (77.9%), musculoskeletal disorder (73.2%), cerebrovascular disease (72.5%), and chronic heart failure (66.2%).

**Table 1:** Patients’ characteristics (*N* = 16,084)

Patients	Age at death, years, mean (SD)	85.1 (7.5)
	65–74 years, n (%)	1539 (9.6%)
	75–84 years	5857 (36.4%)
	85–94 years	6892 (42.9%)
	≥ 95 years	1796 (11.2%)
	Male	7804 (48.5%)
	Female	8280 (51.5%)
Medical care services	Hospitalization, total	14,687 (91.3%)
	Subacute/chronic care hospital	11,378 (70.7%)
	Acute care hospital	8995 (55.9%)
	Home medical care by a physician	3518 (21.9%)
	Ventilation	2852 (17.7%)
	Death at home/long-term care facility	2113 (13.1%)
	Tube feeding	814 (5.1%)
	Hospice	639 (4.0%)
	Dialysis	557 (3.5%)
Long-term care services	Home visiting care	5239 (32.6%)
	Day care	4894 (30.4%)
	Institutional care, total	3779 (23.5%)
	Special nursing home for the elderly	1669 (10.4%)
	Health services facility for the aged	1517 (9.4%)
	Community-based specific facility	394 (2.4%)
	Sanatorium-type medical care facilities	385 (%)
	Group home for dementia	289 (1.8%)
Diseases	Chronic respiratory failure	12,551 (78.0%)
	Hypertension	12,534 (77.9%)
	Musculoskeletal disorder	11,778 (73.2%)
	Cerebrovascular disease	11,659 (72.5%)
	Chronic heart failure	10,640 (66.2%)
	Spinal disease	8396 (52.2%)
	Psychiatric disorder	8041 (50.0%)
	Malignancy	7080 (44.0%)
	Metabolic bone disease	6453 (40.1%)
	Chronic liver disease	6386 (39.7%)
	Dementia	6110 (38.0%)
	Chronic kidney disease	5129 (31.9%)
	Other fracture	3546 (22.0%)
	Benign prostatic hyperplasia	2231 (13.9%)
	Ischemic heart disease	2115 (13.1%)
	Fracture of femur	1964 (12.2%)
	Uncomplicated diabetes mellitus	1926 (12.0%)
	Complicated diabetes mellitus	1686 (10.5%)
	Neurodegenerative disease	1515 (9.4%)
	Fracture of extremities (excluding femur)	531 (3.3%)
	Hematological malignancy	3546 (22.0%)
	Number of diseases Average (minimum–maximum)	7.8 (0–18)

Except where stated, the data are presented as number (percentage). *SD* standard deviation

### Expenditures per patient

Table 2 summarizes the expenditures per patient over the 60 months prior to death. The mean total expenditures per patient for the last 60 months was US $135,851, of which US $87,532 was for medical care and US $48,319 was for long-term care. The mean total expenditures per patient for the last 12 months was US $50,731, of which US $38,054 was for medical care and US $12,677 was for long-term care. Participants whose expenditure was zero throughout the target period were also included in the analysis. There were 5125 patients with zero long-term care expenditures for the last 60 months and 5902 patients with zero such expenditures for the last 12 months.

**Table 2:** End-of-life care expenditures per patient

	Average	SD	Minimum	Maximum
**Expenditures per patient for the 60 months before death (US$)**				
Total of medical and long-term care	135,851	101,556	40	828,497
Total of medical	87,532	83,236	40	806,578
Inpatient	61,925	73,408	40	806,578
Outpatient	25,607	35,118	0	563,884
Total of Long-term care	48,319	68,987	0	314,912
Institutional care	27,217	62,139	0	314,912
Home care	21,102	37,942	0	277,091
**Expenditures per patient for the 12 months before death (US$)**				
Total of medical and long-term care	50,731	31,580	40	463,663
Total of medical	38,054	32,692	40	463,663
Inpatient	31,302	31,938	40	460,031
Outpatient	6752	9650	0	149,329
Total of Long-term care	12,677	16,339	0	66,040
Institutional care	5418	9967	0	60,938
Home care	7259	15,301	0	66,040

*SD* standard deviation

The relationship between individual total of medical and long-term care expenditures for the last 60 months and their cumulative ratio are described as a Pareto chart in Additional file Fig. S4. The cumulative ratio of 10.0% corresponds to the 3.1% percentile rank (rank 510), and the cumulative ratio of 50.0% corresponds to the 24.2% percentile rank (rank 3901).

### Spending trajectories

**Fig. 1:**
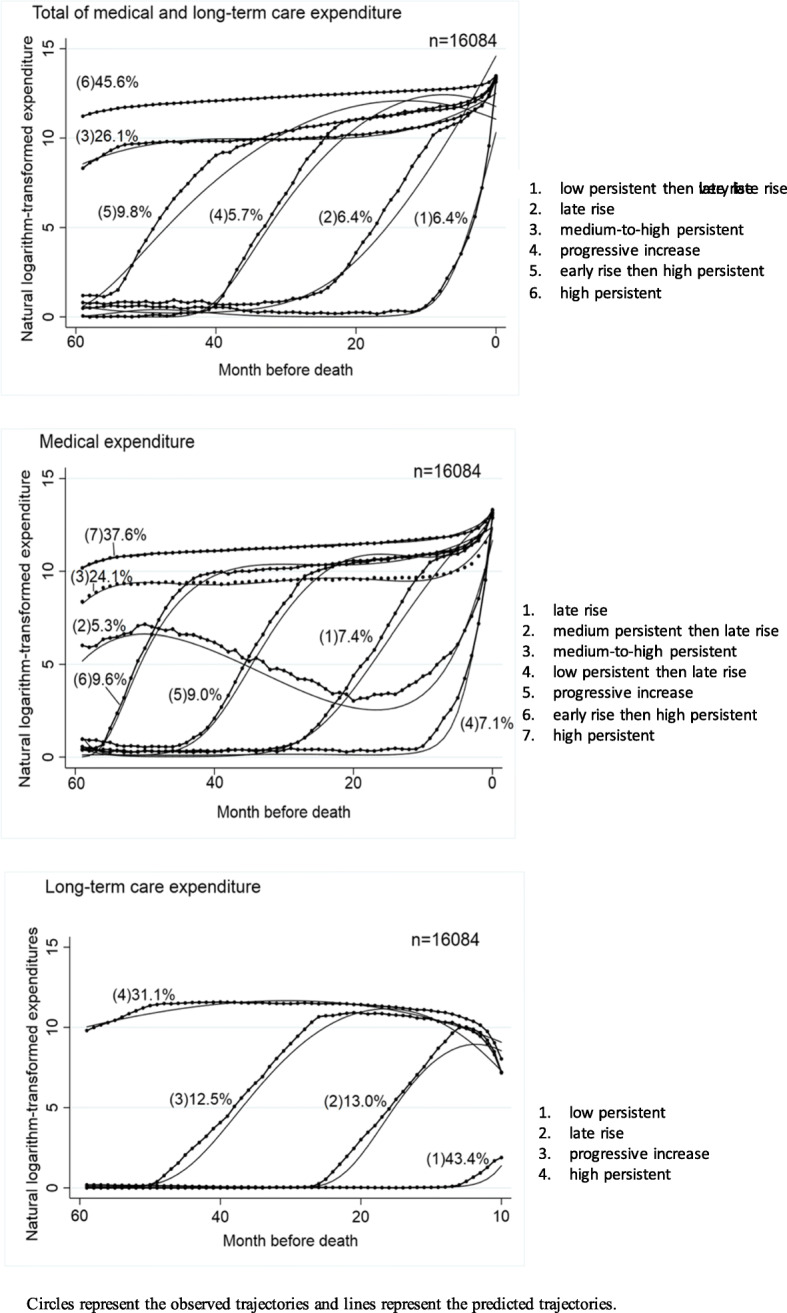
Estimated spending trajectories over 5 years before death. Circles represent the observed trajectories and lines represent the predicted trajectories

The group-based trajectory modeling identified six distinct spending trajectories over the 60-month period for the total expenditures of medical and long-term care combined: high persistent (45.6%), medium-to-high persistent (26.1%), early rise then high persistent (9.8%), late rise (6.4%), low persistent then very late rise (6.4%), and progressive increase (5.7%). There were seven trajectories for medical expenditures: high persistent (37.6%), medium-to-high persistent (24.1%), early rise then high persistent (9.6%), progressive increase (9.0%), late rise (7.4%), low persistent then late rise (7.1%), and medium persistent then late rise (5.3%). There were four trajectories for long-term care expenditures: low persistent (43.4%), high persistent (31.1%), late rise (13.0%), and progressive increase (12.5%). The estimated trajectories and proportions of each groups are shown in Fig. 1.

For the male subgroup, six distinct spending trajectories were identified for the total combined expenditures of medical and long-term care: high persistent (52.7%), medium-to-high persistent (12.9%), progressive increase (9.8%), early rise then high persistent (8.2%), late rise (7.9%), and low persistent then late rise (7.3%). There were seven trajectories for medical expenditures: high persistent (56.8%), early rise then high persistent (10.4%), progressive increase (7.9%), medium persistent then late rise (6.5%), low persistent then progressive increase (6.4%), late rise (6.1%), and low persistent then late rise (5.8%).

There were six trajectories for long-term care expenditures: high persistent (17.9%), early rise then high persistent (9.4%), progressive increase (8.5%), low persistent then progressive increase (9.9%), low persistent then late rise (12.8%), and low persistent (41.5%). The estimated trajectories and proportions of each groups are shown in Additional file Figure S6.

For the female subgroup, four distinct spending trajectories were identified for the combined total expenditures of medical and long-term care: high persistent (60.6%), medium-to-high persistent (22.0%), progressive increase (8.7%), and late rise (8.7%). There were seven trajectories for medical expenditures: high persistent (34.9%), medium-to-high persistent (27.2%), medium persistent then late rise (5.1%), early rise then high persistent (9.4%), progressive increase (9.2%), low persistent then late rise (6.9%), and late rise (7.2%). Moreover,

there were four trajectories for long-term care expenditures: high persistent (43.4%), progressive increase (11.9%), late rise (11.7%), and low persistent (12.5%). The estimated trajectories and proportions of each groups are shown in Additional file Figure S7.

The accuracy of the models was evaluated using the four diagnostic measures and confirmed to be adequate. All the average posterior probabilities of assignment were > 0.7 and the odds of correct classification were > 5.0 for all the groups. The proportions of the sample assigned to each group were confirmed to be sufficiently close to the proportions estimated from the model. The 98% confidence intervals for the estimated proportions were narrow for all the groups. The evaluation of assignment accuracy is described in Additional file S5 Table.

### Characteristics of trajectories

Additional file Tables S8, S9, S10 summarize the end-of-life care expenditures associated with different spending trajectories.

With the total costs for medical and long-term care combined, the highest spending group (high persistent) accounted for 70.6% of the sum of expenditures for all patients in the combined medical and long-term care expenditures, 60.9% in medical expenditures, and 88.2% in long-term care expenditures. With medical expenditures, the highest spending group (high persistent) accounted for 50.0% of the sum of expenditures for all patients in the combined medical and long-term care expenditures, 58.1% in medical expenditures, and 35.4% in long-term care expenditures. With long-term care expenditures, the highest spending group (high persistent) accounted for 47.6% of the sum of expenditures for all patients in the combined medical and long-term care expenditures, 29.3% in medical expenditures, and 80.6% in long-term care expenditures.

Table 3 shows the cross-tabulation of the number of participants assigned to each trajectory group. (A) shows medical care expenditure trajectories versus the total of medical and long-term care trajectories, (B) shows medical versus the total of medical and long-term care trajectories, and (C) shows long-term care versus the total of medical and long-term care trajectories. Across all the combinations of spending categories, the combination of high persistent trajectories contains the highest number of participants, whereas, for the relationship between medical and long-term care trajectories and, the combination of low persistent in long-term care trajectory and high persistent in medical trajectory contained the second-highest number of participants.

**Table 3:** Relationship between trajectories in each expenditure category. Relationship between medical care expenditure trajectories and long-term care trajectories

(A) Relationship between medical care expenditure trajectories and long-term care trajectories								
		Long-term care spending trajectories						
		Low persistent	Late rise	Progressive increase		High persistent		Total
	Late rise	961	53	23		153		1,190
Medical spending trajectories	Medium persistent then late rise	280	77	139		350		846
	Medium-to-high persistent	1,483	452	470		1,467		3,872
	Low persistent then late rise	761	211	41		124		1,137
	Progressive increase	698	216	182		347		1,443
	Early rise then high persistent	724	213	249		366		1,552
	High persistent	2,080	862	903		2,199		6,044
	Total	6,987	2,084	2,007		5,006		
(B) Relationship between medical care expenditure trajectories and the total of medical and long-term care trajectories								
		Total of medical and long-term care spending trajectories						
	Low persistent then late rise	Late rise	Medium-to-high persistent	Progressive increase		Early rise then high persistent	High persistent	Total
Medical spending trajectories	Late rise	961	49	15		15	14	136
	Medium persistent then late rise	59	116	202		4	101	364
	Medium-to-high persistent	0	0	2,239		0	73	1,560
	Low persistent then late rise	7	823	13		168	23	103
	Progressive increase	0	46	26		727	326	318
	Early rise then high persistent	0	0	173		0	1,039	340
	High persistent	0	0	1,529		0	2	4,513
	Total	1,027	1,034	4,197		914	1,578	
(C) Relationship between long-term care expenditure trajectories and total of medical and long-term care trajectories.								
		Total of medical and long-term care spending trajectories						
		Low persistent then late rise	Late rise	Medium-to-high persistent	Progressive increase	Early rise then high persistent	High persistent	Total
Long-term care spending trajectories	Low persistent	1,004	812	2,639	584	840	1,108	6,987
	Late rise	23	218	875	201	272	495	2,084
	Progressive increase	0	2	439	129	423	1,014	2,007
	High persistent	0	2	244	0	43	4,717	5,006
	Total	1027	1034	4197	914	1578	7334	

### Expenditures per patient and monthly time course related to time-to-death

Additional file Figure S11 shows the trend in the monthly costs per patient over the 60 months before death for all the participants and by six trajectories of total of medical and long-term care. Monthly total, total of medical expenditures, and total of inpatient expenditures increased as death approached. However, the long-term care expenditures increased only slightly over this period, and it decreased during the 4 months before death.

### Factors related to trajectory assignment

Patients’ characteristics associated with age, sex, medical service use, and diseases are summarized in Additional file Tables S12, S13, and S14. Additional file Tables S15, S16, and S17 show the odds ratios and 95% confidence intervals obtained from the multinomial logistic regression analyses.

As described in Table S15, with the total expenditures for medical and long-term care combined, compared to the reference group (high persistent trajectory), The explanatory variables with notably high odds ratio were use of hospice in the low persistent then late rise trajectory, malignancy in the late-rise trajectory, and hematological malignancy in the progressive increase trajectory. In particular, the odds ratio was low as a whole in tube feeding and neurodegenerative disease. In the low persistent then late rise trajectory the explanatory variables with particularly low odds ratio were neurodegenerative disease.

As described in Table S16, with the medical care expenditures, compared to the reference group (high persistent trajectory), the explanatory variables with notably high odds ratios were use of hospice in the late rise trajectory, fracture of femur in the medium-to-high persistent trajectory, death at home/long-term care facility in the low persistent then late rise trajectory, and dementia in the early rise then high persistent trajectory. Explanatory variables with particularly low odds ratios included tube feeding, ear disease, and neurodegenerative disease in the late rise trajectory, as well as home medical care by a physician and dialysis in the medium persistent then late-rise trajectory.

As described in Table S17, with the long-term care expenditures, the following associations were observed: compared to the reference group (high persistent trajectory), the explanatory variables with notably high odds ratio were hematological malignancy in the low persistent and the late rise trajectories. The explanatory variables with particularly low odds ratio were home medical care by a physician in the low persistent trajectory, death at home/long-term care facility in the late rise trajectory, dementia, neurodegenerative disease and fracture of the femur in the low persistent trajectory and late rise trajectory.

As for Model 2, the number of diseases was related to the high persistent trajectory in total of medical and long-term care expenditure and medical expenditure; however, a similar trend was not seen with long-term care.

## Discussion

In the trajectories of medical and long-term care expenditures for the 5 years before death, the most frequent pattern was the high-persistent trajectory. It was speculated that most patients began to experience a marked increase in end-of-life costs more than 5 years before death. Factors associated with the high-persistent trajectory were associated with chronic illnesses, various organ failures, neurodegenerative diseases, and fractures. Patients with malignancy tended to have higher spending for medical care than long-term care and to show relatively rapid increases in expenditure before death. Patients with high-persistent spending trajectories in medical expenditures belonged to high spending trajectories and low spending trajectories in long-term care expenditures almost equally. In contrast, most patients with high-persistent spending trajectories in long-term care expenditures belonged to high spending trajectories in medical expenditures.

### End-of-life medical expenditures in Japan

The Japanese Ministry of Health, Labor and Welfare estimated that the lifetime medical expenditure per capita in Japan in 2015 was US $290,000 (27 million Japanese yen), of which 50% was expended for patients aged > 70 years [29]. In this study, the medical expenditures per patient during 60 months before death (mean age = 85.1 ± 7.5 years) was US $87,532; this corresponded to approximately 30% of the lifetime medical expenditures in Japan. The per capita total medical and long-term care expenditures in this study was US $135,851 during the 60 months before death and US $50,731 during the 12 months before death. A study by French and colleagues, which compared end-of-life healthcare expenditures internationally in nine countries, reported that total medical and long-term care expenditures during the 12 months before death were US $80,094 in the USA, US $63,476 in the Netherlands, US $62,672 in Denmark, US $52,742 in Germany, and US $20,892 in Taiwan (only medical expenditure data are provided for the rest of the world) [14]. Our study was limited to people aged ≥70 years (at the time of death); thus, simple comparisons are impossible because of differences in conditions such as measurement and exchange rate. However, we posit that healthcare expenditures in older adults in Japan were not significantly dissociated from those in other developed countries.

### Trajectories of medical and long-term care expenditures during the final 5 years before death

The most frequently observed trajectory for medical and long-term care expenditures during the 5 years before death was the high-persistent trajectory, which accounted for 45.6% of the patients, 70.6% of the total expenditures, 60.9% of the total medical expenditures, 88.2% of the total long-term care expenditures, and consumed most healthcare resources. This result was consistent with that of Davis and colleagues’ study of medical expenditures during the final year before death for patients in the USA, which also reported that the most frequent trajectory was high persistent—accounting for the highest proportion of total expenditures [11]. These findings suggest that high end-of-life healthcare demand can start at least 5 years before death, concerning both medical and long-term care. Further study is warranted, using data covering a longer period.

As described in Additional Figures S6 and S7, we performed a similar analysis for gender subcategories. We found that women demonstrated slower increases in total medical spending than men. Meanwhile, medical spending was more likely to be persistently high in men, while long-term care constituted a high a proportion of high persistent trajectory in women. This result was consistent with Multiple Multinomial Logistic Regression. These findings may be attributed to the fact that women have a longer life expectancy than men and are more likely to demonstrate decreases in their ADL near end of life.

### Relationship between end-of-life medical and long-term care expenditure trajectories

In the cross-tabulation of the distribution of trajectories by cost category to which each participant belongs, the combination of high persistent trajectories and high persistent trajectories was most frequent, in the total of medical and long-term care and medical spending. For the comparisons of medical versus long-term care trajectories, the combination of low persistent in long-term care trajectory and high persistent in medical trajectory were equally frequent. These results suggest that patients with sustained high long-term care expenditures have a higher demand for both medical and long-term care and, consequently, a higher total for combined medical and long-term care expenditures. In contrast, about half the patients with sustained high medical expenditures had higher expenditures for long-term care; but a major part of health expenditures for the other half was medical care.

### Factors associated with the expenditure trajectories

Davis and colleagues noted that the high-persistent trajectory for Medicare expenditures was associated with multiple chronic illnesses rather than specific illnesses [10]. Focusing on explanatory variables suggestive of a relatively strong association in multiple multinomial logistic regression analysis, the factors associated with high-persistent trajectory were similar to that in previous studies: multimorbidity and multiple chronic organ damage. However, in addition, neurodegenerative disease and fracture of the femur and other extremities were also associated with high-persistent trajectory in each category, and the use of medical services and tube feeding were also associated in a similar manner. Our study also suggested a consistent relationship between specific diseases and trajectory. The results of multiple multinomial logistic regression analyses suggest that malignancy is positively associated with late-rise trajectory in combined medical and long-term care expenditures (hematological malignancy is associated with progressive trajectory) and negatively associated with the assignment to high-persistent trajectory. In addition, in long-term care expenditures, it was associated with the low persistent trajectory. Therefore, as indicated by previous studies and empirical findings [4, 5, 30], ,malignancy is a process in which deterioration of function and increased demand for healthcare are occurring relatively immediately before death; and medical care and not long-term care plays a major role in end-of-life care for malignancy.

A study conducted by Stabenau and colleagues on functional disability 1 year before hospice admission in a group-based trajectory modeling analysis indicated that neurodegenerative disease tended to be associated with a sustained decline in physical function, whereas cancer did not [31], which was similar to the current results. Further research should compare changes in actual physical function with both medical and long-term care expenditures.

In addition, dementia was related to the assignment to high-persistent trajectories in combined medical and long-term care expenditures; whereas it was related to early rise then high persistent in medical expenditures, and to progressive in long-term care. Therefore, we conclude that end-of-life healthcare spending for dementia increases relatively slowly over the five-year period before death, compared to conditions with multiple chronic diseases, organ damage, and neurodegenerative diseases; however, both medical and long-term care are required.

### Strengths

In Japan, most people aged 65 years or older are covered by the National Health Insurance or the Late-stage Elderly Health Insurance: in 2016, the proportions covered were 73.6% of those aged 65–75 years and 97.9% of those aged over 75 years [32, 33] The long-term care insurance system was introduced in Japan in 2000, and the amount of use has been increasing annually. Although there are financial challenges, the long-term care demands of the elderly in Japan are largely covered by long-term care insurance [19]. Our study is one of the few studies that examined the trajectories of end-of-life expenditures concerning both medical and long-term care using a highly comprehensive database that addressed older adults. Therefore, the results of this study may be useful for visualizing the amount of end-of-life care needed by elderly patients.

### Limitations

This study had some limitations. Information about diseases was extracted from the coding of medical claims. However, this coding may not correspond to patients’ actual diseases because the system only records conditions that were actually treated during the period; that is, drugs that are often prescribed to older patients for off-label use [34] cannot be detected and therefore the diseases on claims data may not always reflect all diseases—however, it is likely there would be few such cases. Meanwhile, a significant limitation should be noted regarding the explanatory variables for disease: the time of onset could not be identified, and so the association with the outcome is limited. Moreover, there was no available information about confounding factors that may affect expenditures, such as caregiver [35, 36], ,socioeconomic status [37, 38], ,and preferences of physician [39]. The associations between healthcare expenditures and explanatory variables in this study may therefore be underestimated; however, this does not negate the findings. Third, this study was based on a database of one prefecture in Japan and thus has external validity limitations. Larger studies based on national databases should be conducted in the future. Furthermore, the results may not be generalizable to countries other than Japan. However, because some results mirrored those found in other countries, such as the United States [10, 11] the results of this study may have universal findings.

### Implications

There are two main implications. First, this study found that most targets of end-of-life care are a group with highly sustained healthcare demands during the five years before death, but not patients with increased healthcare demands in the short pre-mortem period. These results may require a change in thinking among end-of-life care providers.

Advanced care planning discussions, along with appropriate palliative care, are essential to enhance end-of-life care [40]. However, the difficulty and uncertainty of prognosis, especially for non-cancer diseases, is a communication challenge between healthcare providers and patients in providing end-of-life care and is a major cause of difficulties in providing end-of-life care [6]. Even in cancers, which are said to be relatively prognostic, there are prognosis difficulties [41]. Einav and colleagues suggested that the medical expenditures incurred by patients with high mortality rates are small and that it is difficult to predict patients with high end-of-life expenditures in advance [42]. The current results suggest that end-of-life trajectories and their onset may be somewhat predictable in cancer, but that end-of-life starting points may not be clear in patients with multimodality or neurodegenerative diseases. Consequently, our result may support clinicians’ empirical realization about end-of-life prognosis.

Therefore, healthcare providers may be forced to reconsider the timing appropriate to discuss advanced care planning, whether effective advanced care planning is feasible, and what is effective for improving the quality of end-of-life care. For example, in a qualitative study, Bern-Klug and colleagues noted the value of ﻿social interactions related to the healthcare and dying status of patients to amend patients’ and their families’ end-of-life experience [43]. In end-of-life care, providers are required not only to provide formal medical care but also to provide individualized care. Although our study and other previous studies indicated that multimorbidity plays a key role in end-of-life functional decline, the increased number of patients with multimorbidity implies a variety of comorbid disease combinations and may imply a diversity of end-of-life disease states. Therefore, providers of end-of-life care may need to refine the points of communication with patients—with outcomes of improved end-of-life patient experience—to be more personalized rather than categorized per disease trajectories.

Second, this study suggest that another indication is the need to consider long-term interventions in the resource allocation of end-of-life care, which involves at least 5 years of perseverance in both healthcare and long-term care expenditures. Our results indicated the sustained high costs over the 5 years before death, as well as the concentration on medical resources just before death. Under the current Japanese remuneration system, remuneration for assessing palliative care is only adapted for the short period immediately before death from malignancy. However, the results of this study suggest the usefulness of evaluating the quality of end-of-life care from a chronic perspective in coordination with care services.

## Conclusions

With the trajectories of medical and long-term care expenditures for the 5 years before death, the most frequent pattern was the high-persistent trajectory. Factors associated with the high-persistent trajectory were chronic illnesses, various organ failures, neurodegenerative diseases, and fractures. Patients with malignancy tended to have a higher demand for medical care spending than for long-term care and to show relatively rapid increases before death. Patients with high-persistent spending trajectories in medical expenditures belonged to high spending trajectories and low spending trajectories in long-term care expenditures almost equally; in contrast, most patients with high-persistent spending trajectories in long-term care expenditures belonged to high spending trajectories in medical expenditures. New insights into end-of-life healthcare needs may lead to a change in thinking in considering the delivery of end-of-life care and the distribution of end-of-life care resources. Although this study infers that for most patients, end-of-life expenditures began to markedly increase more than 5 years before death, there is room to examine the beginning of this increase. Furthermore, it is necessary to explore the factors associated with high and low individual expenditures within each trajectory group. To overcome the limitations of this study and to obtain unknown findings, there is room for further investigation, analysis using nationwide data over a longer period, and more detailed analysis of high expenditure groups.

## Supplementary Information

**Additional file 1: Table S1.** Correspondence between Japanese public healthcare services and System of Health Accounts (SHA) 2011. **Table S2.** Correspondence between the comorbidities and International Classification of Diseases (ICD-10) codes. **Text S3.** The group-based trajectory model. **Figure S4.** Total of medical and long-term care expenditures and cumulative ratio. **Table S5.** Evaluation of assignment accuracy. **Figure S6.** Estimated spending trajectories of male over 5 years before death. **Figure S7.** Estimated spending trajectories of female over 5 years before death. **Table S8.** End-of-life care expenditures associated with combined medical and long-term care spending trajectories. **Table S9.** End-of-life care expenditures associated with the medical spending trajectories. **Table S10.** End-of-life care expenditures associated with the long-term care spending trajectories. **Figure S11**. Trends of end-of-life care expenditures per patient per month during the 60 months. **Table S12.** Patient characteristics associated with the combined medical and long-term care spending trajectories. **Table S13.** Patient characteristics associated with the medical spending trajectories. **Table S14.** Patient characteristics associated with the long-term care spending trajectories. **Table S15.** Multiple multinomial logistic regression analyses for combined medical and long-term care spending trajectories. **Table S16.** Multiple multinomial logistic regression analyses for medical spending trajectories. **Table S17.** Multiple multinomial logistic regression analyses for long-term care spending trajectories.

## Data Availability

Our data are not available for publication owing to an ethical restriction. Specifically, we are restricted from making the minimal dataset publicly available owing to regulations set the Federation of National Health Insurance Associations. However, data are available from the Federation of National Health Insurance Associations research group for researchers who meet the criteria for access to these confidential data. Requests to access the data should be submitted to Professor Yuichi Imanaka, Department of Healthcare Economics and Quality Management, School of Public Health, Graduate School of Medicine, Kyoto University, Yoshida Konoe-cho, Sakyo-ku, Kyoto 606–8501, Japan. Email: heqm-office@umin.ac.jp.

## Abbreviations

OECD: Organization for Economic Co-operation and Development
SHA: System of Health Accounts
