# Supplementary material for: Trajectories of end-of-life medical and long-term care expenditures for older adults in Japan: retrospective longitudinal study using a large-scale linked database of medical and long-term care claims
Source: BMC Geriatr. 2021 Jun 30;21:403. doi: 10.1186/s12877-021-02215-9 (PMC8243899; doi:10.1186/s12877-021-02215-9)
Supplement: Supplementary file 1 — Additional file 1: Table S1. Correspondence between Japanese public healthcare services and System of Health Accounts (SHA) 2011. Table S2. Correspondence between the comorbidities and International Classification of Diseases (ICD-10) codes. Text S3. The group-based trajectory model. Figure S4. Total of medical and long-term care expenditures and cumulative ratio. Table S5. Evaluation of assignment accuracy. Figure S6. Estimated spending trajectories of male over 5 years before death. Figure S7. Estimated spending trajectories of female over 5 years before death. Table S8. End-of-life care expenditures associated with combined medical and long-term care spending trajectories. Table S9. End-of-life care expenditures associated with the medical spending trajectories. Table S10. End-of-life care expenditures associated with the long-term care spending trajectories. Figure S11. Trends of end-of-life care expenditures per patient per month during the 60 months. Table S12. Patient characteristics associated with the combined medical and long-term care spending trajectories. Table S13. Patient characteristics associated with the medical spending trajectories. Table S14. Patient characteristics associated with the long-term care spending trajectories. Table S15. Multiple multinomial logistic regression analyses for combined medical and long-term care spending trajectories. Table S16. Multiple multinomial logistic regression analyses for medical spending trajectories. Table S17. Multiple multinomial logistic regression analyses for long-term care spending trajectories. [file 12877_2021_2215_MOESM1_ESM.xlsx › Text_S3R4.docx]

**Text S3. The Group-Based Trajectory Model**

**Trajectory Identification**

Let $Y_{i}$ = ({$y_{i1}$,$y_{i2}$,_・・・・_, $y_{iT}$}) denote the longitudinal sequence of costs for each individual period *i* of T periods, $P\left( Y_{i} \right)$the unconditional probability of $Y_{i}$, $P^{j}\left( Y_{i} \right)$the probability of $Y_{i}$ given membership of group *j*, and $\pi_{j}$ the probability of a randomly chosen population member belonging to group *j*. Then

$$P\left( Y_{i} \right)=\sum_{j}^{J} \pi_{j}P^{j}\left( Y_{i} \right)$$

With an assumption of conditional independence for the sequential realizations of the elements of $Y_{i}$ and $y_{it}$over the *T* periods of measurement,

$$P^{j}\left( Y_{i} \right)=\prod_{1}^{T} P^{j}\left( Y_{it} \right)$$

where $P^{j}\left( Y_{it} \right)$ is the probability distribution function of $y_{it}$, given membership in group *j*.

The likelihood for all N individuals is

$$L=\prod_{1}^{N} P\left( Y_{i} \right)$$

For the censored normal distribution, and for a cubic trajectory, the model can be calculated from

$y_{it}^{*}$= $\beta_{0}^{j}+\beta_{1}^{j}x_{it}+\beta_{2}^{j}x_{it}^{2}+\beta_{3}^{j}x_{it}^{3}+\varepsilon_{it}$

where $x_{it}$ is time and $\beta_{0}^{j}$, $\beta_{1}^{j}$, $\beta_{2}^{j}$, and $\beta_{3}^{j}$ are parameters that determine the shape of the polynomial.

**Evaluation of the Adequacy of the Models**

The accuracy of the models was evaluated using four diagnostic measures: the average posterior probability of assignment for each group (0.7 or higher), the odds of correct classification (5.0 or higher), the proportion of a sample assigned to a certain group is close to the proportion estimated from the model, and 98% confidence intervals for the estimated proportions.

The posterior probability of individual *i*’s membership of group j is denoted by$\hat{P}\left( j | Y_{i} \right)$.This cannot be calculated directly from the model’s parameter estimates; however, it is possible to calculate a related probability, the probability of $Y_{i}$ assuming membership of group *j*, denoted by $\hat{P}\left( Y_{i} | j \right)$. This is calculated as follows:

$$\hat{P}\left( j | Y_{i} \right)=\frac{\hat{P}\left( Y_{i} | j \right)\hat{\pi}_{j}}{\sum_{j}^{J} \hat{P}\left( Y_{i} | j \right)\hat{\pi}_{j}}$$

The odds of correct classification for group *j* (${OCC}_{j}$) can be calculated from

$${OCC}_{j}=\frac{{{AvePP}_{j}}/\left( 1-{AvePP}_{j} \right)}{{\hat{\pi}_{j}}/{(1}-\hat{\pi}_{j})}$$

where AvePP is the average posterior probability.

**REFERENCE**

1. Nagin D. *Group-Based Modeling of Development*. Harvard University Press; 2005.
